# Indigenous communities’ perceptions reveal threats and management options of wild edible plants in semiarid lands of northwestern Kenya

**DOI:** 10.1186/s13002-023-00584-6

**Published:** 2023-05-04

**Authors:** Wyclife Agumba Oluoch, Cory Whitney, Céline Termote, Christian Borgemeister, Christine B. Schmitt

**Affiliations:** 1 Center for Development Research (ZEF), University of Bonn, Bonn, Germany; 2 Institute of Crop Science and Resource Conservation (INRES), Horticultural Sciences, University of Bonn, Bonn, Germany; 3 Alliance of Bioversity International and International Center for Tropical Agriculture (CIAT), Nairobi, Kenya; 4 Geography Section, University of Passau, Passau, Germany

**Keywords:** Conservation, Sustainable use, Wild food plants, Integrated participatory approach, Field survey, Focus group discussion, Local knowledge, Kenya

## Abstract

**Background:**

Understanding how local communities perceive threats and management options of wild edible plants (WEPs) is essential in developing their conservation strategies and action plans. Due to their multiple use values, including nutrition, medicinal, construction, and cultural as well as biotic and abiotic pressures, WEPs are exposed to overexploitation, especially within arid and semiarid lands, and hence the need to manage and conserve them. We demonstrate how an understanding of indigenous communities’ perceptions could be achieved through an integrated participatory approach involving focus group discussions (FGDs) and field plot surveys.

**Methods:**

We conducted three FGDs between October 2020 and April 2021 within three community units in northwestern Kenya with different socioeconomic and environmental characteristics. We subsequently surveyed 240 field plots of size 1 ha each to assess threats facing WEPs within a 5 km buffer radius in every study community. We compared ranks of threats and management options across community units.

**Results:**

Rankings of threats and management options differed across the three study communities. We obtained strong positive linear relationships between field and FGD rankings of threats facing WEPs. Climate change, overstocking, overharvesting, and invasive species were the highest-ranked threats. Mitigation of climate change, local knowledge preservation, selection, propagation, processing, and marketing of WEPs ranked high among possible management options irrespective of the socioeconomic and environmental characteristics of the community unit.

**Conclusions:**

Our approach emphasizes the relevance of leveraging indigenous communities’ perceptions and conducting field plot surveys to assess threats and management options for WEPs. Evaluating the effectiveness and cost–benefit implications of implementing the highly ranked management options could help determine potentially suitable habitats of the WEPs for conservation and management purposes, especially for priority WEPs.

**Supplementary Information:**

The online version contains supplementary material available at 10.1186/s13002-023-00584-6.

## Background

Wild edible plants (WEPs) are ‘safety nets’ for many communities during lean seasons [1–3] and in times of conflict [4, 5]. They have been essential assets in the fight against malnutrition and hunger in many societies [6–8] and of benefit to modern communities and in the future [9, 10]. However, WEPs have witnessed continued localized habitat destruction and overexploitation [11, 12], attributable to various anthropogenic and natural factors [13, 14]. Such factors compromise the sustainable use of WEPs as safety nets for many communities across the globe [15].

Within Africa, threats to WEPs pose challenges to about 80% of the rural populations that derive food from the wild [16]. The threats inhibit the optimal regeneration of WEPs and their use as food by such communities [17, 18]. While some threats have adverse effects on the local abundance of WEPs, changes in lifestyle and consumption patterns, among other socioeconomic and cultural reasons, also explain the declining use of WEPs [19, 20]. The impacts from such threats are primarily felt by poor rural people [16, 21], thus negatively affecting the role of WEPs as ‘safety nets’ for rural African populations vulnerable to malnutrition and hunger [22, 23].

Turkana County in northwestern Kenya is one of the affected regions in Africa. It is inhabited by the Turkana people, among others, whose traditional livelihood strategy is nomadic pastoralism [24, 25]. Accordingly, their primary diet comprises animal products like meat, milk, and blood. They derive plant-based vitamins and herbal medicines primarily from WEPs [20, 26, 27]. Some communities have diversified their livelihood strategies into trade, such as the sale of *Aloe vera* [28, 29], honey harvesting [25, 30], artisanal gold mining [31], poultry keeping [32], basket weaving [33–35], hide processing [36], local brewing [37], fishing [38, 39], and crop cultivation [25, 40].

Of the 47 counties in Kenya, Turkana County has the highest poverty and malnutrition rates [41]. Only 3.2% of its population hold food stocks that can last more than one month [42]. Against the national poverty headcount rate of 36.1%, it has the highest poverty rate of 79.4% (about 80% of Turkana people are considered poor) [42]. The county also has the highest food poverty rate at 66.1%, compared to the national average of 32% [42]. With WEPs known to aid in food and nutritional security [43], assessing their threats and management options could be a significant step in sustainably utilizing them in such a setting as Turkana County.

Turkana people have relied on locally constituted management methods like seasonal grazing (via migration with livestock) and clear designation grazing fields [44]. These, however, could not be sufficient in countering contemporary threats, including those of anthropogenic climate change. Managing valuable resources such as WEPs for sustainable use is crucial to local communities. We define ‘sustainable use’ as the case when WEPs are harvested within the limits of their carrying capacity for self-renewal and the manner of harvest does not degrade the environment in other ways [45].

We sought to understand the threats and management options that could aid the sustainable use of WEPs in northwestern Kenya. To achieve this, we used an integrated participatory approach to combine FGDs results with field plot surveys guided by three research questions: (i) Which threats do WEPs face in Turkana County, and how do they vary across different socioeconomic and environmental settings? (ii) How do indigenous communities’ perceptions of these threats compare with field survey results? (iii) What are possible effective management options and how do they differ across socioeconomic and environmental settings?

## Materials and methods

### Study area description

**Fig. 1:**
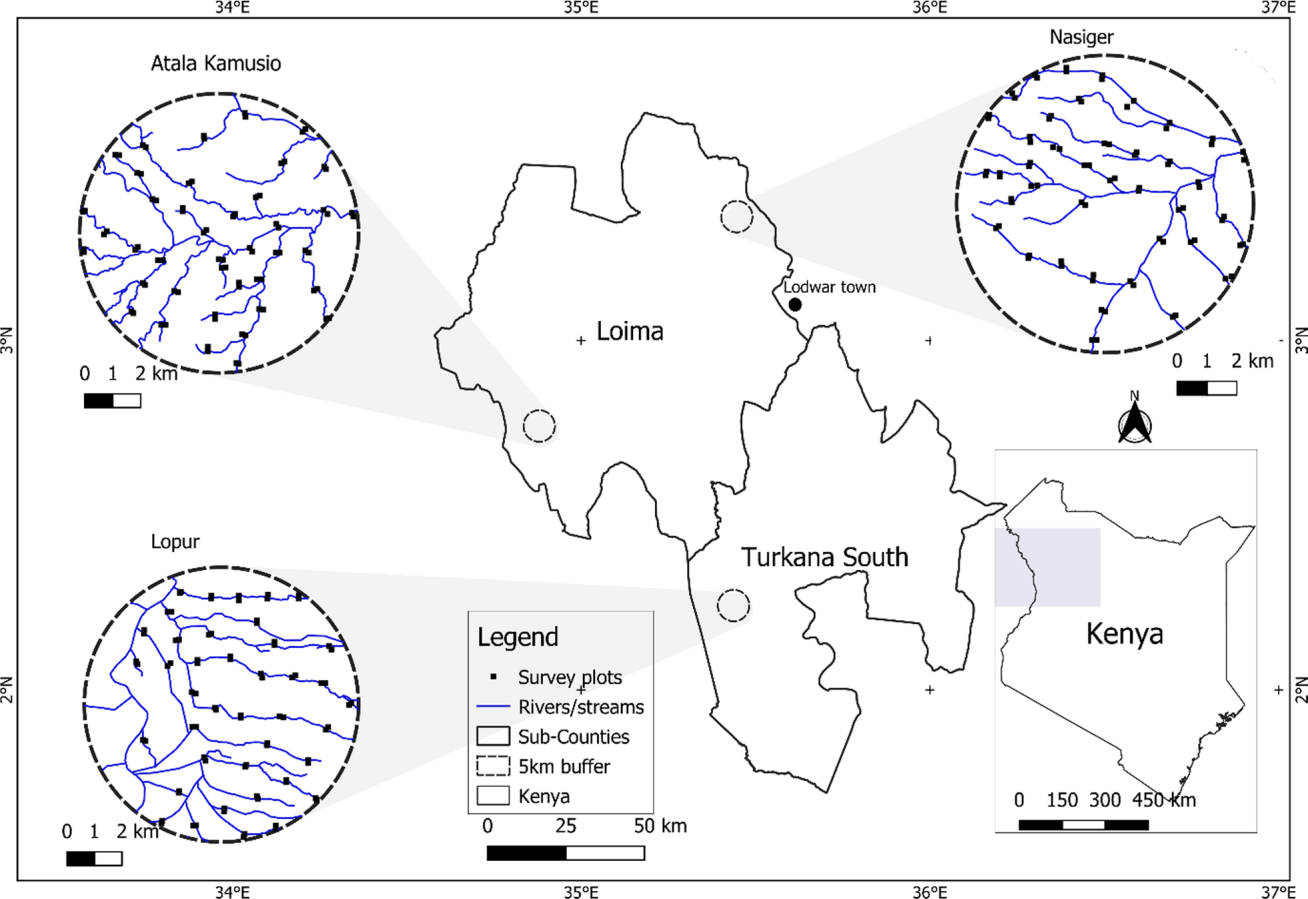
Study area map showing the distribution of the study plots within the three community units of Nasiger, Atala Kamusio, and Lopur within Loima and Turkana South sub-counties of Turkana County, Kenya

We conducted the study in three community health units (Nasiger, Atala Kamusio, and Lopur), reflecting the socioeconomic and environmental differences in Turkana County (Fig. 1). A community health unit, hereafter called a community unit, is a designated geographical zone with approximately 1000 households and served by ten community health volunteers and one health extension worker [46]. Nasiger community is located in the dry plains about 40 km north of Lodwar town, the headquarter of Turkana County. It receives an annual rainfall of about 166 mm (average 1981—2022) [47]. The vegetation consists of scanty scrubs with occasional trees along the riparian areas (normally dry riverbeds) [44]. According to the Food Economy Group, the community unit falls under the Turkana Central Pastoral livelihood zone, an “exceptionally hot, dry, and arid environment” [48].

Atala Kamusio community is situated in the Turkana Border Pastoral livelihood zone [48], about 100 km west of Lodwar town (Fig. 1). The landscape undulates between mid- and lowland elevations, and woody and shrubby plants dominate the landscape [44]. It receives an annual rainfall of 371 mm (average 1981—2022) [47]. The Lopur community is in the Turkwel Riverine-Agro Pastoral livelihood zone [48], about 118 km south of Lodwar town (Fig. 1), along the only permanent river in Turkana County, the Turkwel River. The area receives 327 mm of rainfall per year (average 1981—2022) [47] and has intensive crop cultivation with irrigation water from the river [49]. Inhabitants grow crops such as maize, beans, tomatoes, and pawpaw and keep livestock such as cattle, sheep, goats, camels, and donkeys [49, 50].

### Data collection

We obtained threats and management options data on WEPs from the literature and discussed these with each of the three community units during FGDs. We also conducted field observations of threats. The research activities were carried out from October 2020 to April 2021.

#### Extraction of threats and management option categories from the literature

We extracted threats and management options for WEPs from published literature using a snow-ball sampling approach [51]. We went through as many literature sources as possible (*n* = 23) that featured either threat or management reports. The list of threats and management options with their corresponding reviewed sources are given in Additional file 1: Table s1 and Table s3, respectively. We also obtained threat categories from the threats classification scheme version 3.2 by the International Union for Conservation of Nature (IUCN) [52]. We then went through all threat categories in the obtained literature. We grouped categories referring to similar threats, e.g., by placing “expansion of agriculture” and “expansion of agricultural land” into the same category (Additional file 1: Table s2).

#### Focus group discussions

We held FGDs with 14 adults (age > = 18 years) participants in each of the three study community units [53]. With the help of local administrators, we included participants in the study who were involved in the WEPs value chain, including harvesting, use, and conservation efforts. They included, among other community members, village elders, community health volunteers, church leaders, community nutritionists, public health officers, community health workers, and teachers.

Village elders, for example, oversee matters concerning the use and conservation of community resources, including WEPs. Local administrators maintain peace and ensure adherence to rules, such as settling disputes whenever they arise, including those concerning WEPs. They are also the main entry points to the communities for government and non-government programs. Teachers instill knowledge in the young generation in school settings, including nutrition skills that could involve the use of WEPs. Health workers, such as health extension officers, nutritionists, public health officers, and community health volunteers at the community level, support the improvement of the health and well-being of local people, including advocating for the use of WEPs in their diets. Lastly, other members of the FGDs were drawn from residents who participated in harvesting and use of WEPs for food and medicine, among others. We thus considered all the participants very resourceful in discussing threats and management options for WEPs.

We selected three priority woody WEPs, i.e., *Salvadora persica*, *Ziziphus mauritiana*, and* Balanites rotundifolia*, considered priority [53] due to their high use values in the region for detailed FGDs with the local participants. We opted for woody species as their longer lifespan in the field implied that participants interacted with them more and could discuss them more exhaustively. Further, the trees were also present in the field during our plot surveys.

We commenced every FGD by allowing participants to free list and discuss threats facing the three priority woody WEPs. We then consensually co-grouped the listed threats into the nine pre-defined (cf. 2.2.1) categories with the participants. We added a tenth category for all mentioned threats that were not in our nine pre-selected categories (Additional file 1: Table s2). We did preference ranking [54–56] that involved asking the participants to score each of the ten threat categories on a scale of 10 (threat of greatest concern) to 1 (threat of least concern) according to their perceived magnitude of effects on the three priority woody WEPs. We gave each participant 10 white circular pieces of cardboard, and they raised a card after concluding the discussion on each threat category. We took note of the number on the raised cardboard by each participant. We repeated that for all ten threat categories as we expounded on the indicators under each threat category. We ranked management options in the same manner.

#### Field observations of threats to woody wild edible plants

We obtained geographic coordinates of the FGD venue in each of the three community units using a handheld global positioning system (GPS). Treating this as the central point of the community unit, we created a virtual buffer zone of a five-km radius (Fig. 1) as buffers, within which we traced all rivers/streams using QGIS software [57] and Google Earth base layers. Though there were no distinct boundary maps of the community units to help derive the centers, the local communities considered our FGD sites central. None of our five-km buffers overlapped with neighboring community units. Based on prior informal discussions with local administrators, we assumed that the participants, and other community members in general, resided within that radius, and their scored threats would be represented within that spatial extent. We then generated 40 random survey plots and established 100 m by 100 m (1 ha) plots at each point along the digitized streams such that no two plots were closer than 1 km (Fig. 1). For every random riverine plot, we generated a corresponding off-riverine plot at least 100 m from the river bank. That resulted in 80 survey plots per community unit and 240 survey plots for the three community units. We chose to include ‘riverine’ as a factor since our study area was largely arid. We assumed occasional higher relative moisture levels confined within riverine areas could explain some variations. Both *S. persica* and *Z. mauritiana* have also been reported to prefer riverine sites [58–60].

Using nine of our ten threat categories (we dropped ‘climate change’ as it was impractical to observe indicators of climate change in a single field visit), we scored observable threats to WEPs in each survey plot. Each threat category could receive a score between 1 (least threat) and 9 (highest threat). Scoring was based on the consensus of the threat categories by three observers (two trained research assistants and the corresponding author). Observed indicators of threats included fire scars to denote fire threat, over-browsed seedlings/lower branches of priority woody WEPs to denote overstocking/overgrazing, and plowed land to characterize agriculture expansion threat, among others that were obtained from FGDs (Additional file 1: Table s2).

### Data analysis

We calculated score-sums [54, 56] for all threat and management scores from all participants to obtain an overall ranking of each threat and management category for all community units combined and individually. We then tested for differences in the scores within and between community units for management options and scores from threats we observed from field plot surveys. We ran the test using nonparametric *friedmanAlignedRanksTest* and *friedmanAlignedRanksPost* functions in the *scmamp* [61] package version 0.3.2 in R [62] version 4.2.2. The test is well-suited for nonparametric, non-normally distributed, and ranked or ordinal data. To compare FGD and field plot survey ranking of threats to woody WEPs, we ran pairwise correlation tests on the resulting rankings.

## Results

### General description of FGD participants

Participants in the FGDs were individuals knowledgeable about WEPs (cf. 2.2.2) within Turkana County, Kenya. Their representation is summarized in Table 1.

**Table 1:** Proportion of participants in the FGDs

Participants	Number included in the study	Proportion (%)
Village elders	7	17
Chiefs/assistants chiefs	5	12
Teachers	5	12
Health workers	8	19
Nutritionists	3	7
Public health officers	1	2
Other community members	13	31
Total	42	100

Table 1 indicates that many of the participants, 31% (*n* = 13), were ordinary community members, followed by health workers, village elders, and a public health officer. 60% (*n* = 25) of the participants were female, 40% (*n* = 17) were male. The participants were considered diverse and knowledgeable enough to give detailed discussions on the WEPs needed for the study.

### Threats to woody wild edible plants from focus group discussions

Climate change, invasive species, and overstocking/overgrazing ranked highest among the threats facing priority woody WEPs according to scores by FGD participants (Table 2). We observed similar patterns in the Nasiger and Atala Kamusio community units but not in Lopur. Agriculture expansion, selective harvesting/overharvesting, and overstocking/overgrazing were ranked the highest here. We then tested for possible differences in threat scores.

**Table 2:** Rank summaries of threat categories in each community unit and across all community units combined (Nasiger, Atala Kamusio, and Lopur) in Turkana County, Kenya

Threat categories	Nasiger		Atala Kamusio		Lopur		All communities	
	Score	Rank	Score	Rank	Score	Rank	Score	Rank
Climate change	117	1	111	1	57	8	285	1
Invasive species	88	2	104	2	89	6	281	2
Overstocking/overgrazing	65	3	93	3	102	3	260	3
Selective harvesting/overharvesting	61	4	76	4	110	2	247	4
Fuelwood collection/charcoal burning	43	5	69	5	97	5	209	5
Agricultural expansion	23	9	38	7	130	1	191	6
Uncontrolled fire	40	6	25	9	101	4	166	7
Infrastructural development	31	7	62	6	72	7	165	8
Pests and diseases	30	8	33	8	41	9	104	9
Others	16	10	19	10	17	10	52	10

Each score represents the sum of scores from cards raised by 14 participants in the focus group discussion. For all communities, we summed the scores from each community unit. The rank column indicates the order of the scores

**Fig. 2:**
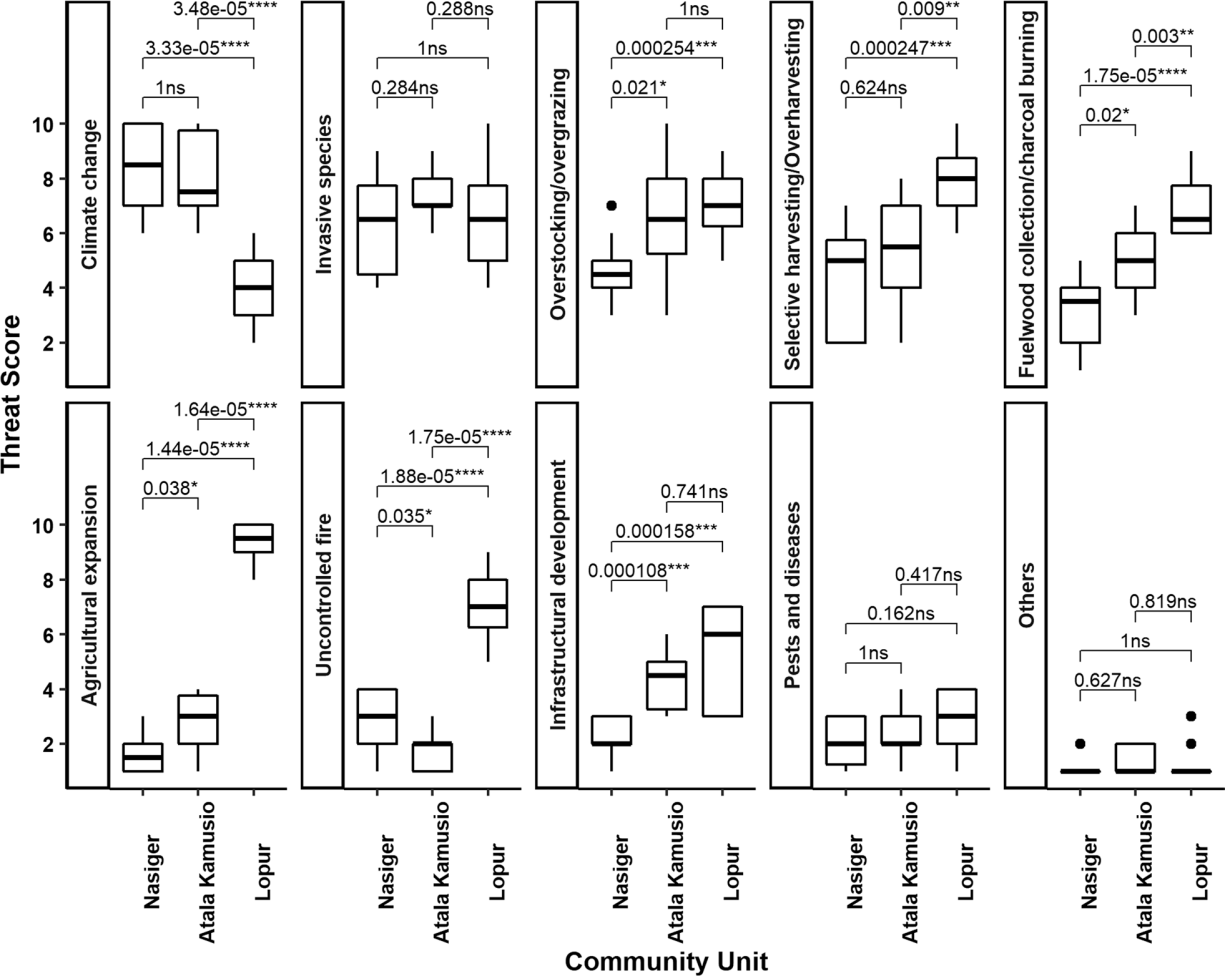
Comparison of scores on threats across the three study community units in Turkana County, Kenya. ns, *, **, ***, and **** represent not significant, significant at α = 5%, 1%, 0.1% and 0.01%, respectively

At least one community unit was significantly different (α < 5%) from the other(s) in the ranking of each threat category except for invasive species, pests and diseases, and others (Fig. 2).

### Threats to woody wild edible plants from field observations

We observed no significant differences in threat scores between riverine and off-riverine field survey plots; hence, we formed a composite of the two datasets resulting in 80 survey plots per community unit. Overstocking/overgrazing, invasive species, and selective harvesting/overharvesting were the top three threats we observed in the field at Nasiger (Table 3). At Atala Kamusio, top three threats were overstocking/overgrazing, selective harvesting/overharvesting, and fuelwood collection/charcoal burning. Agricultural expansion was the top-ranked threat to WEPs at Lopur, followed by invasive species and uncontrolled fire (Table 3). In field plot observations, overstocking/overgrazing was the highest-ranked threat, followed by selective harvesting/overharvesting and invasive species (Table 3). The same threats were identified in the FGDs.

**Table 3:** Sum of scores and ranks of threat categories from field plot observations (*n* = 80) in three community units (Nasiger, Atala Kamusio, and Lopur and all combined) in Turkana County, Kenya

Threat categories	Nasiger		Atala Kamusio		Lopur		All communities	
	Score	Rank	Score	Rank	Score	Rank	Score	Rank
Overstocking/overgrazing	581	1[2]	635	1[2]	615	4[3]	1831	1[2]
Selective harvesting/overharvesting	471	3[3]	557	2[3]	442	5[2]	1470	2[3]
Invasive species	483	2[1]	332	4[1]	628	2[6]	1443	3[1]
Fuelwood collection/charcoal burning	269	4[4]	448	3[4]	387	6[5]	1104	4[4]
Agricultural expansion	80	9[8]	168	6[6]	658	1[1]	906	5[5]
Uncontrolled fire	105	7[5]	160	7[8]	617	3[4]	882	6[6]
Infrastructural development	168	6[6]	154	8[5]	282	7[7]	604	7[7]
Pests and diseases	197	5[7]	204	5[7]	129	8[8]	530	8[8]
Others	102	8[9]	83	9[9]	82	9[9]	267	9[9]

Ranks drawn from FGDs, after dropping climate change threat (see Table 1), are indicated in brackets [] for comparison

**Fig. 3:**
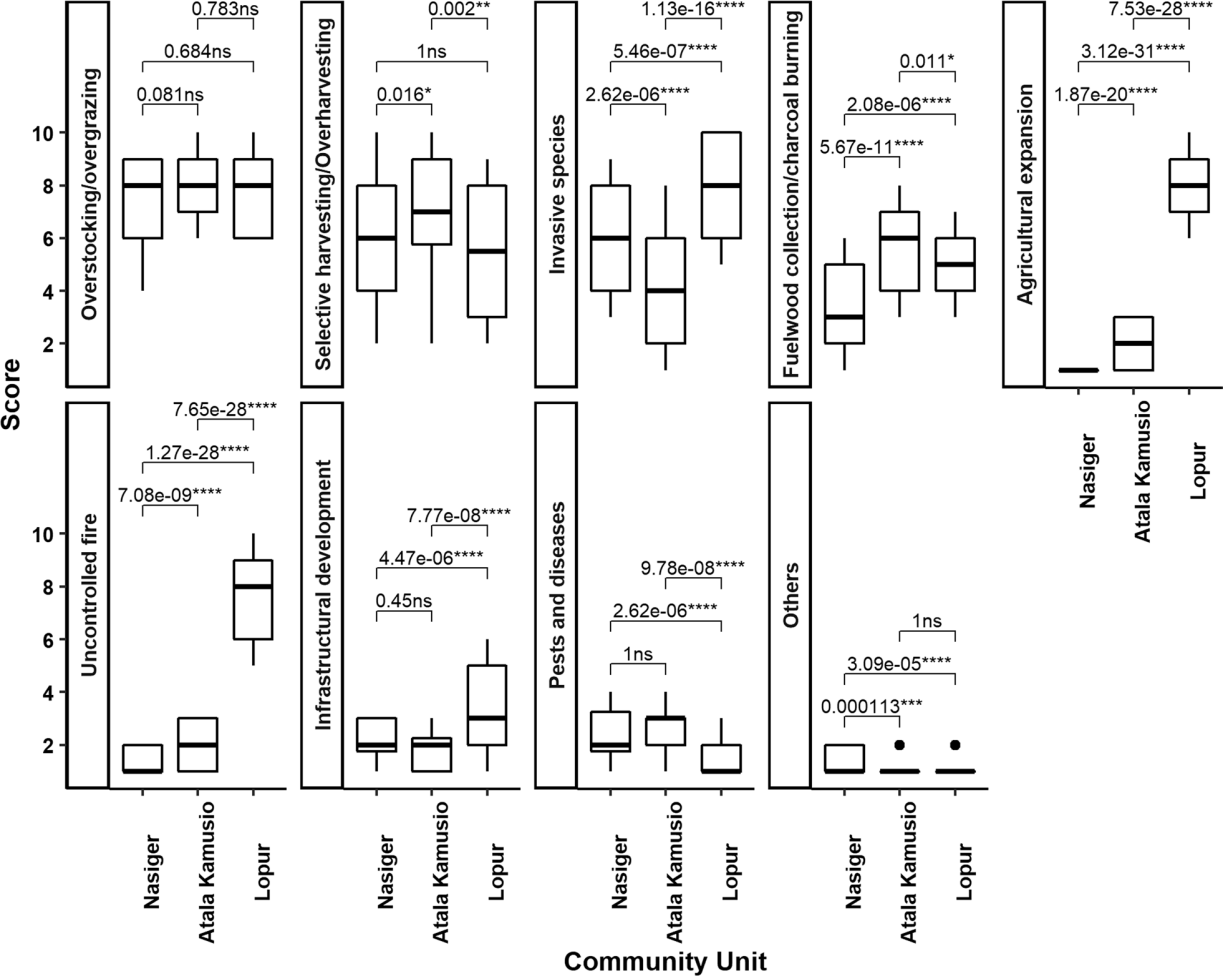
Comparison of rankings of threat categories observed in the field across three study community units in Turkana County, Kenya. ns, *, **, ***, and **** represent not significant, significance at α = 5%, 1%, 0.1% and 0.01%, respectively

Our rankings of threat categories facing woody WEPs from the field plots surveys varied significantly among the study community units (Fig. 3). We, however, observed some similarities in the rankings, as were the cases for overstocking/overgrazing at all communities, selective harvesting/overharvesting at Atala Kamusio and Lopur, infrastructural development at Nasiger and Lopur, pests and diseases at Nasiger and Lopur, and others at Nasiger and Atala Kamusio.

**Fig. 4:**
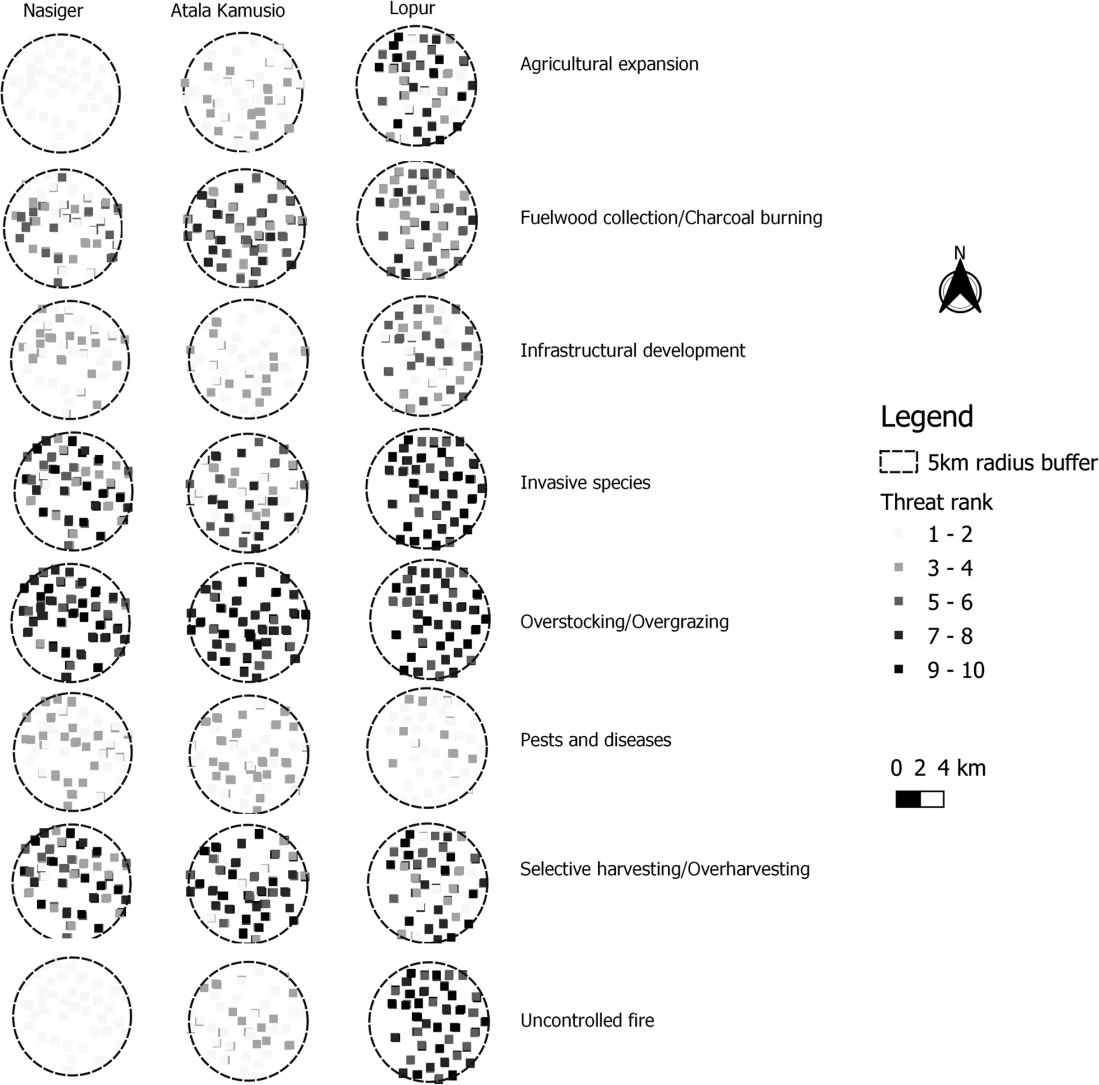
Map showing the variation in the ranking of threat categories facing priority woody wild edible plants within three study community units (Nasiger, Atala Kamusio, and Lopur) in Turkana County, Kenya. We did not include the ‘Others’ category in the figure as it had negligible rankings, while climate change indicators were not assessed in the field

To spatially visualize variations in scores among threats facing priority WEPs in all the 240 surveyed plots and community units, we developed a graduated gray scale map (Fig. 4). For example, overstocking/overgrazing ranked similarly high in almost all three community units. At the same time, the agricultural expansion was least in Nasiger and highest in Lopur.

**Fig. 5:**
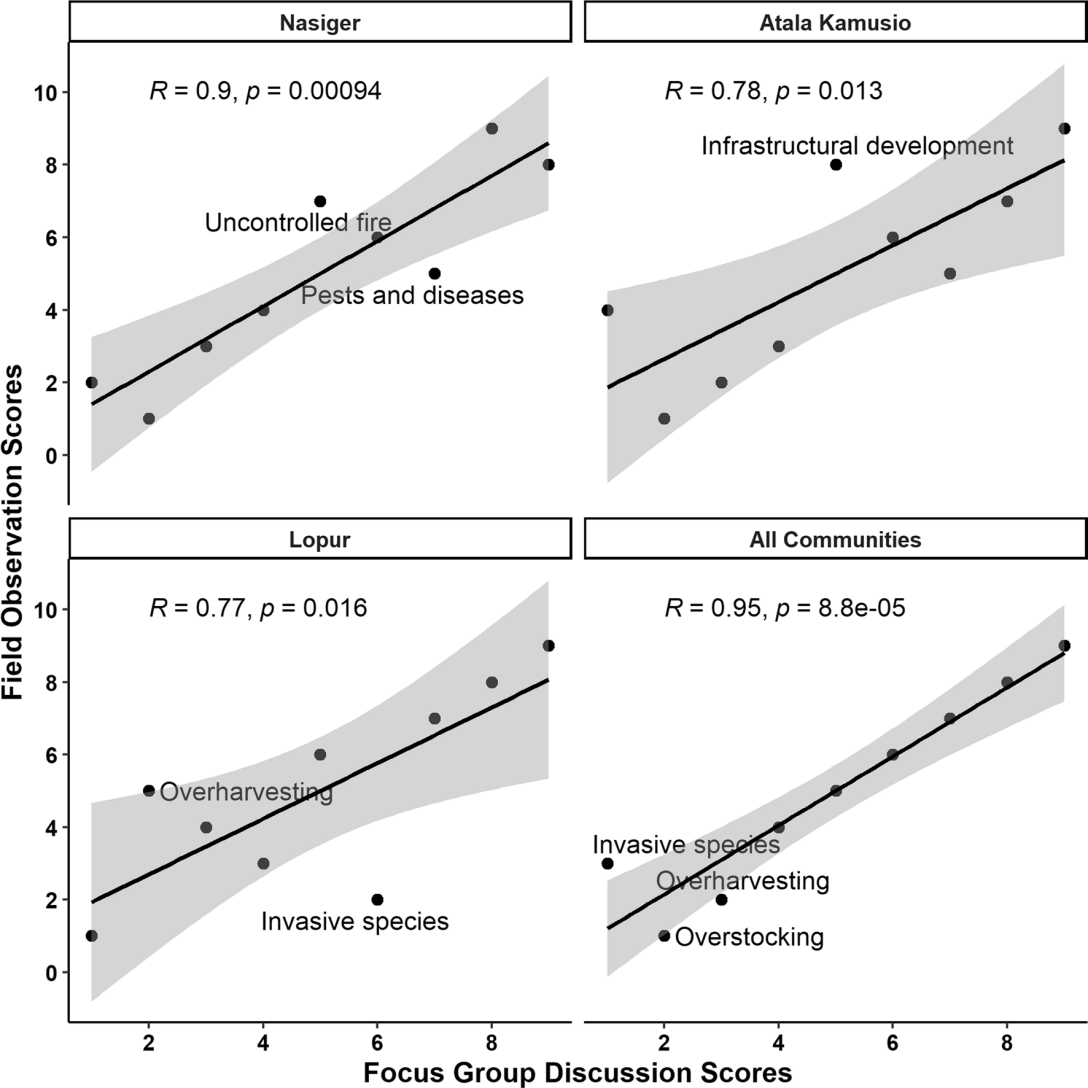
Association between focus group discussion and field plot survey ranking of studied threat categories (except climate change) facing wild edible plants within Nasiger, Atala Kamusio, and Lopur community units in Turkana County, Kenya. The gray margin area around the best line of fit (black line) represents the 95% confidence interval. Points outside that margin are labeled by their threat category names

Figure 5 shows how the scores for threats (except climate change) in FGDs are associated with that of field plot surveys. Strong positive linear associations existed between FGD rankings and field plot survey rankings of threats facing priority woody WEPs in Turkana County, similar to the one-to-one ranking in Table 3.

### Management options for threats to priority woody wild edible plants

Overall, the three study communities mentioned mitigation of climate change, preservation of local knowledge about WEPs, and selection, propagation, processing, and marketing as the highest-ranked management options for threats facing WEPs (Table 4). At least two of these top three management options appeared among the top three for each community unit individually. However, no two community units attained similarity for the top-ranked management option per community unit. Nasiger, Atala Kamusio, and Lopur community units ranked control harvesting for food and fodder, cultivating WEPs, and mitigating climate change as their top-ranked management options, respectively. We thus checked for possible similarities and differences in the ranked management options.

**Table 4:** Scores and ranks of management options by participants (*n* = 14 in each community unit and *n* = 42 for all the three community units combined) for threats to wild edible plants in Turkana County, Kenya

Management categories	Nasiger		Atala Kamusio		Lopur		All communities	
	Score	Rank	Score	Rank	Score	Rank	Score	Rank
Mitigate climate change	113	3	138	2	133	1	384	1
Preserve local knowledge about the WEPs	115	2	137	3	130	5	382	2
Selection, propagation, processing and marketing	110	4	136	4	132	2	378	3
Cultivate WEPs	100	6	139	1	131	3	370	4
Control harvesting for food and fodder	122	1	121	6	119	7	362	5
Prohibit charcoal burning	86	7	136	4	131	3	353	6
Assess nutrition and toxicity	110	4	107	8	114	8	331	7
Create public awareness on WEPs	72	9	109	7	124	6	305	8
Conserve in sacred areas	80	8	102	9	76	10	258	9
Establish protected areas	62	10	46	10	102	9	210	10
Monitor and inventor	38	12	35	12	43	11	116	11
Others (home gardens, pruning and pollarding, enhance participatory planning, alternative livelihood for local people)	39	11	38	11	31	12	108	12

Scoring was done by each participant at a scale of 1 (least ranked management option) to 12 (highest-ranked management option) and summed for every community unit individually and all community units combined

**Fig. 6:**
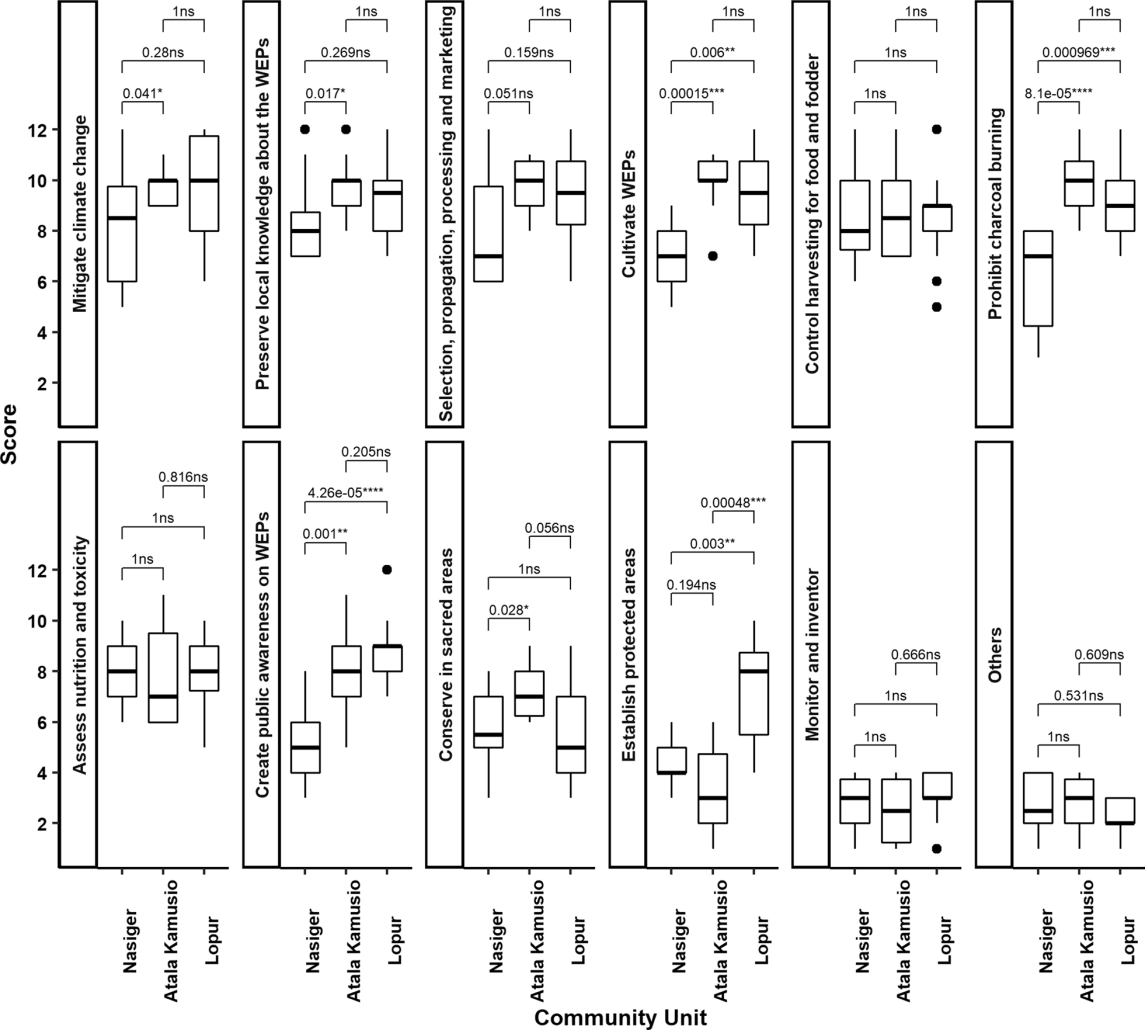
Mean comparisons of management options in three community units in Turkana County, Kenya. ns, *, **, ***, and **** represent not significant, significant at α = 5%, 1%, 0.1%, and 0.01%, respectively

Out of the 12 scored management options, there were seven with significant differences in at least two compared community units (Fig. 6). The four management options, control harvesting for food and fodder, assess nutrition and toxicity, monitor and inventor, and others, ranked similarly across the community units, suggesting commonality in how the FGD participants from the study communities perceived the management options.

## Discussion

We assessed threats facing priority woody WEPs from local community perspectives involving FGDs and field plot surveys in an integrated participatory approach. We also assessed management options with the potential to counter the adverse effects of these threats from the point of view of FGD participants. From the FGDs, we most importantly found climate change, invasive species, and overstocking/overgrazing to be among the highest-ranking threats facing WEPs in Turkana County. Our findings from the field plot surveys revealed that overstocking/overgrazing, selective harvesting/overharvesting, and invasive species were the top-ranking threats. Field plot surveys and FGD rankings of threats showed strong positive linear relationships. We found mitigation of climate change, preservation of local knowledge, and selection, propagation, processing, and marketing to be the highest-ranking management options for the priority woody WEPs.

Our FGDs and field observations results on threats correspond to those from similar studies conducted in southern Ethiopia that put agricultural land expansion, fuelwood collection, uncontrolled fire setting, overgrazing, and overharvesting as highly ranked threats to WEPs [17, 63–65]. The different socioeconomic and environmental settings of the studied community units can explain the observed differences in the scoring of threats facing WEPs: For example, inhabitants of the three community units derived their livelihoods differently. While livestock keeping was predominant in Nasiger and Atala Kamusio, crop farming dominated in Lopur [48]. The extensive irrigated croplands astride the banks of River Turkwel in Lopur partly explained why this community scored the threat of agricultural expansion highest. Efforts by the Kenyan government to expand agricultural land for irrigated crop farming since 2015 [48] could jeopardize the future of WEPs in the region.

In terms of invasive species, although receiving average to high scores across the communities, no differences among the community units could be detected. The dominant invasive species in northwestern Kenya, *Prosopis juliflora* [66, 67], was perceived by all three community units as a threat to the priority woody WEPs. This species was highlighted by the FGDs as highly invasive, a fodder to livestock although known to destroy teeth of goats, and is used for charcoal to try and manage its spread. We also observed the species in the field surveys.

Climate change was perceived by the FGD participants in terms of a range of indicators that they experienced in the region (Additional file 1: Table s2). We acknowledge that these could be subjective and that structured scientific investigations could help reveal the extent of the impact of climate change or variability on WEPs in the region. WEPs have the potential to cushion a community against the negative impacts of climate change [68–70], climate change can also threaten their sustainable use [14, 64]. Further, overstocking/overgrazing could also inhibit optimal production of WEPs while at the same time inhibiting the regeneration potential as the seedlings or propagules get stampeded, overgrazed/overbrowsed [71, 72].

Our results on the potential management options for priority woody WEPs indicated that mitigation of climate change, preservation of local knowledge about WEPs, and carrying out selection, propagation, processing, and marketing of WEPs in the region were perceived as plausible. While the communities called for documenting local knowledge about the WEPs and passing that knowledge to current generations, they also understood that climate change should be mitigated and that scientists could help in selecting WEPs, propagating them on a large scale, processing/improving on traditional preservation methods to add value, and availing them in the market for sustainable income generation.

Implementing management options such as mitigation of climate change [73, 74], controlling harvesting [75], establishing protected areas [76–78], and nutritional and genetic profiling [79] have been proposed to protect WEPs, and some places implemented with notable successes [80, 81]. In particular, Feyssa [81] in Ethiopia showed how important indigenous knowledge and its intergenerational transfer could aid the management and conservation of WEPs. Marketing has also been reported as a potential management strategy of WEPs elsewhere [82] because communities that derive an income from the sale of fruits from WEPs will also consider them more valuable and worthy of conservation. Moreover, propagation and cultivation are also reported elsewhere as potential ways to use WEPs sustainably [83–85].

In more recent work, Borelli [86] emphasized the need for an integrated conservation approach to better manage WEPs. This would entail cooperation across sectors and diverse stakeholders in the WEP’s value chain(s). Indeed, we noted that local communities knew the threats facing their WEPs, as indicated by a strong positive linear correlation with our field plot survey scores, and should, accordingly, be integrated into the formulation of WEP management options. Their voice in the implementation of management options should be borne in mind by scientific communities and policymakers alike since they have used their management options to sustainably utilize their resources throughout history.

Among the possible management options mentioned by FGD participants, some could be implemented through local community initiatives, while others would require interventions from external bodies. For instance, the conservation of WEPs in sacred areas (cemeteries, churches, cultural gathering sites), controlling harvesting for food and fodder, cultivation of WEPs, regulation of charcoal burning, and preservation of local knowledge about WEPs could fit within local community action plans (personal communication from FGD participant). On the other hand, the assessment of nutritional value and toxicity, the establishment of protected areas, selection, propagation, processing, and marketing require external intervention but with local collaboration. Some measures, such as raising public awareness about the benefits of WEPs, mitigation of climate change, and monitoring and inventorying WEPs, can only be achieved by closely engaging with local communities, policymakers, and any actors attempting to influence the management of WEPs. Involving local communities in implementing any management option is imperative.

We understand that cost implications always play a big role in implementing any management options for threats facing biodiversity [87]. However, it is beyond the scope of our study to address the question of cost implications in deploying any of the management options to ensure sustainability in the conservation efforts of WEPs. It is important to map the extent of potentially suitable habitats for the WEPs so that conservation and management options can be implemented site-specific. How future climate change scenarios might exacerbate the already existing threats would also be important to determine moving forward.

Even though these findings agreed well with most studies on threats to biodiversity across the region, it is important to note that the relative significance varied with environmental and socioeconomic gradients at local scales. Local differences in threats and management options are therefore worth considering in developing sustainable management solutions for WEPs to bring them back into dietary diversification programs sustainably [14, 88].

## Conclusion

Climate change, invasive species, and overstocking/overgrazing threaten the sustainable use of WEPs in Turkana County, Kenya. How threats are perceived to affect WEPs depends on socioeconomic and environmental gradients across communities. Our integrated participatory approach, combining local community perceptions and field plot assessments, revealed close links, but some threats were ranked strikingly differently across the three study community units.

Across all the study communities, the most plausible management options for the WEPs were mitigation of climate change, preservation of local knowledge, and selection, propagation, processing, and marketing. We propose a detailed cost–benefit analysis of the assessed management options, bringing on-board all stakeholders in the WEP value chain, which should be a prerequisite before conservation plans are implemented. It is also important to establish the extent of the suitable habitats of the WEPs. Such an overview could improve the success of conservation and management interventions.

## Supplementary Information

**Additional file 1: Supplementary Table 1**. Threats facing wild edible plants or biodiversity in general mentioned in 23 literature sources and used to guide development of 10 threat categories for both focus group discussions and field plot surveys in Turkana County, Kenya.** Supplementary Table 2**. Indicators derived from the focus group discussions on threats facing priority woody wild edible plants in Turkana County, Kenya. The same indicators were used in field plot surveys except for climate change.** Supplementary Table 3**. Management options for wild edible plants mentioned in 9 literature sources and used to guide development of management categories for focus group discussions in selected community units within Turkana County, Kenya.

## Data Availability

The datasets generated and/or analyzed during the current study are available in the GitHub repository, https://github.com/Wycology/wep_threats_management.

## Abbreviations

FGD: Focus group discussion
GPS: Global positioning system
IUCN: International Union for Conservation of Nature
WEP: Wild edible plant
