# Supplementary material for: Indigenous communities’ perceptions reveal threats and management options of wild edible plants in semiarid lands of northwestern Kenya
Source: J Ethnobiol Ethnomed. 2023 May 4;19:13. doi: 10.1186/s13002-023-00584-6 (PMC10161424; doi:10.1186/s13002-023-00584-6)
Supplement: Supplementary file 1 — Additional file 1: Supplementary Table 1. Threats facing wild edible plants or biodiversity in general mentioned in 23 literature sources and used to guide development of 10 threat categories for both focus group discussions and field plot surveys in Turkana County, Kenya. Supplementary Table 2. Indicators derived from the focus group discussions on threats facing priority woody wild edible plants in Turkana County, Kenya. The same indicators were used in field plot surveys except for climate change. Supplementary Table 3. Management options for wild edible plants mentioned in 9 literature sources and used to guide development of management categories for focus group discussions in selected community units within Turkana County, Kenya. [file 13002_2023_584_MOESM1_ESM.pdf]

**Supplementary Table 1** Threats facing wild edible plants or biodiversity in general mentioned in 23 literature sources and used to guide development of 10 threat categories for both focus group discussions and field plot surveys in Turkana County, Kenya

| Number | The threats cited | Reviewed Source |
|---|---|---|
| 1 | Overgrazing, Agricultural land expansion, Over-harvesting, Uncontrolled fire setting, Roads and home construction, Fodder and fuelwood collection | [1] |
| 2 | Construction and tools, Grazing, Charcoal, Agricultural expansions, Firewood, | [2] |
| 3 | Agricultural land expansion, Over-grazing, Over-harvesting, Uncontrolled fire setting, Fuelwood collection | [3] |
| 4 | Habitat conversion due to agricultural expansion, overgrazing, other problems | [4] |
| 5 | Agricultural expansion, Overgrazing, Fuelwood collection | [5] |
| 6 | Expansion of agriculture, Fire hazards, Overgrazing, Construction of new road, Drought, Collection of firewood and construction materials | [6] |
| 7 | Inconsiderate and overexploitation by “outsiders”, Destruction of EWPs by wild animals such as elephants | [7] |
| 8 | Overgrazing/over browsing, removal of woody plants for different purposes (e.g. firewood and charcoal production, building and construction, fencing materials etc), Human settlement, Agricultural expansion, Burning forests | [8] |

| Number | The threats cited | Reviewed Source |
| --- | --- | --- |
| 9 | Agricultural expansion, Fire, Fuelwood collection, Over-stocking, overgrazing, Selective harvesting | [9] |
| 10 | Agricultural land expansion, Uncontrolled fire setting, Fuelwood collection, Overgrazing, Overharvesting | [10] |
| 11 | Land use change (expansion of agriculture land), Developmental activities (road construction and urbanization), Habitat destruction (Timber harvest, Fuelwood collection, and wildfire), Drought, Overharversting, Overgrazing | [11] |
| 12 | Human population pressure, Land use change, Poverty/hunger, Tribal conflicts, Adverse climate change, Overexploitation of resources, Restriction of mobility, Lack of alternative, High livestock population, Expansion of agriculture. | [12] |
| 13 | Wildfire, Deliberate burning – mostly to get pasture grasses regenerated and control animal exo-parasites like ticks, Deforestation, Bush clearing | [13] |
| 14 | Agriculture, Introducing exotic species, Overgrazing, Construction, Charcoal making, Extended dry seasons | [14] |
| 15 | Agricultural land expansion, Lack of culture of planting, Selective harvesting for other use (house construction, farm | [15] |

| Number | The threats cited | Reviewed Source |
|---|---|---|
| | implements, household utensils), Drought/shortage of rainfall, Fuelwood collection, Grazing pasture, Wildfire | |
| 16 | Increase in human population, The increasing logging for charcoal and timber, The heavy harvesting of plants (especially bark, roots and stems) for medicinal use, bush fires, all of which they perceive as leading to soil erosion, drought, and hunger | [16] |
| 17 | Fuel wood collection, Drought, Selective harvesting, Over-stocking/grazing, Agricultural expansion, Fire hazards | [17] |
| 18 | Land use change, Direct exploitation, Climate change, Pollution, Others, Wild fires, Pests | [18] |
| 19 | Habitat destruction, Land use change, Over-grazing, Over-harvesting, Invasive species | [19] |
| 20 | Agricultural expansion, Fire, Fuelwood collection, Selective harvesting | [20] |
| 21 | Fire, Agricultural expansion, Deforestation, Free grazing, Fuelwood, Herbicides, Construction, Settlement, Other (farm and household tool) | [21] |
| 22 | Agricultural expansion, Construction, Fuelwood collection and charcoal making, Overgrazing, Fire | [22] |
| 23 | Land clearing, Fire, grazing, wood exploitation, fruits/flowers harvest, leaves harvest, back exploitation, | [23] |

| Number | The threats cited | Reviewed Source |
|---|---|---|
| | charcoal, pests and diseases, drought, ageing, loss soil fertility | |

**Supplementary Table 2** Indicators derived from the focus group discussions on threats facing priority woody wild edible plants in Turkana County, Kenya. The same indicators were used in field plot surveys except for climate change

| Number | Threat category | Threat indicators mentioned by FGD participants |
|---|---|---|
| 1 | Climate change | Severe wilting or drying, *Prosopis juliflora*, loss of surface water sources, extreme precipitation, extreme wind events, reducing population of WEPs, emergence of non-native invasive species, overabundance of some native species |
| 2 | Invasive species | Invasive *Prosopis juliflora*, emergence of some harmful microbial organisms |
| 3 | Overstocking/overgrazing | Scars of browsed seedlings, browsed branches, cutting of branches for feeding livestock, sedentary ranching |
| 4 | Selective harvesting/Overharvesting | Cut stems/branches, stumps identifiable as of WEPs, severely debarked stems, digging of roots of WEPs |
| 5 | Fuelwood collection/charcoal burning | Active or used charcoal smoking kiln, Cut stems awaiting charcoal burning, people collecting fuel wood, level of charcoal business in the area |
| 6 | Agricultural expansion | Ploughed land, crops or remains of crops, cleared land awaiting ploughing |
| 7 | Uncontrolled fire | Fire scars on stems/branches/leaves, burnt piece of land, fire from charcoal burning, fire from honey harvesting, |
| 8 | Infrastructural development | New developed structures such as institutions and roads, establishing of homesteads |
| 9 | Pests and diseases | Signs of pests on the plant such as locusts, signs of pest damage on leaves, signs of fungal or diseased parts of WEPs |
| 10 | Others | Squirrel/woodpecker holes on trunks of WEPs, destruction of land following war/battlefield, riverbank erosion, and sedimentation, use of herbicides and pesticides, pollution from touristic activities, opportunistic harvesting of branches by tourists along the roads |

**Supplementary Table 3** Management options for wild edible plants mentioned in 9 literature sources and used to guide development of management categories for focus group discussions in selected community units within Turkana County, Kenya

| Number | Management options cited | Reviewed Source |
|---|---|---|
| 1 | Cultivation and marketing | [1] |
| 2 | Home gardens, sacred compounds | [2] |
| 3 | Cultivation and marketing | [3] |
| 4 | Domestication and cultivation, studying nutritional values, propagation techniques, marketing and value addition | [8] |
| 5 | *In-situ* conservation methods like planting in the form of fences and protected pasture land in different worship areas, and in farm margins | [10] |
| 6 | Create awareness of WEPs use by indigenous people, Value indigenous knowledge, Enhance participatory planning and implementation of projects, Alternative livelihoods for local people | [24] |
| 7 | Increasing awareness, agroforestry, garden trees | [25] |
| 8 | Planting around home gardens, Pruning, Pollarding, Fencing, Protected by culture | [22] |
| 9 | Adoption of longer intervals between fires | [23] |

## References


1. Abbasi AM, Khan MA, Khan N, Shah MH. Ethnobotanical survey of medicinally important wild edible fruits species used by tribal communities of Lesser Himalayas-Pakistan. J Ethnopharmacol. Elsevier; 2013;148:528–36.

2. Alemayehu G, Asfaw Z, Kelbessa E. Plant diversity and ethnobotany in Berehet District, North Shewa Zone of Amhara Region (Ethiopia) with emphasis on wild edible plants. J Med Plants Stud. 2015;3:93–105.

3. Ali-Shtayeh MS, Jamous RM, Al-Shafie' JH, Elgharabah WA, Kherfan FA, Qarariah KH, et al. Traditional knowledge of wild edible plants used in Palestine (Northern West Bank): A comparative study. J Ethnobiol Ethnomed [Internet]. 2008;4:13. Available from: https://doi.org/10.1186/1746-4269-4-13

4. Asfaw Z. The future of wild food plants in southern Ethiopia: ecosystem conservation coupled with enhancement of the roles of key social groups. Int Symp Underutilized Plants Food Secur Nutr Income Sustain Dev 806. 2008. p. 701–8.

5. Ashagre M, Asfaw Z, Kelbessa E. Ethnobotanical study of wild edible plants in Burji District, Segan Area Zone of Southern Nations, Nationalities and Peoples Region (SNNPR), Ethiopia. J Ethnobiol Ethnomed. 2016;12.

6. Assefa A, Abebe T. Wild edible trees and shrubs in the semi-arid lowlands of southern Ethiopia. J Sci Dev. 2011;1:5–19.

7. Badimo D, Lepetu J, Teketay D. Utilization of edible wild plants and their contribution to household income in Gweta Village, central Botswana. Afr J Food Sci Technol. 2015;6:220–8.

8. Bahru T, Asfaw Z, Demissew S. Wild edible plants: sustainable use and management by indigenous communities in and the buffer area of Awash national park, Ethiopia. SINET Ethiop J Sci. 2013;36:93–108.

9. Balemie K, Kebebew F. Ethnobotanical study of wild edible plants in Derashe and Kucha Districts, South Ethiopia. J Ethnobiol Ethnomed. 2006;2:1–9.

10. Berihun T, Molla E. Study on the diversity and use of wild edible plants in Bullen District Northwest Ethiopia. J Bot. Hindawi Limited; 2017;2017:10.

11. Duguma HT. Wild edible plant nutritional contribution and consumer perception in Ethiopia. Int J Food Sci. Hindawi; 2020;2020:16.

12. Feyssa DH, Njoka JT, Asfaw Z, Nyangito MM. Uses and management of Ximenia americana, Olacaceae in semi-arid East Shewa, Ethiopia. Pakistan J Bot. Pakistan Botanical Society; 2012;44:1177–84.

13. Guinand Y, Lemessa D. Wild food plants in Southern Ethiopia: Reflections on the role of 'famine foods' at a time of drought. UNDP-EUE F. Mission Report, Addis Ababa. 2000.

14. Kidane L, Kejela A. Food security and environment conservation through sustainable use of wild and semi-wild edible plants: a case study in Berek Natural Forest, Oromia special zone, Ethiopia. Agric Food Secur. Springer; 2021;10:1–16.

15. Kidane B, van der Maesen LJG, Asfaw Z, Sosef MSM, van Andel T. Wild and semi-wild leafy vegetables used by the Maale and Ari ethnic communities in southern Ethiopia. Genet Resour Crop Evol [Internet]. 2015;62:221–34. Available from: https://doi.org/10.1007/s10722-014-0147-9

16. N'Danikou S, Achigan-Dako EG, Wong JLG. Eliciting Local Values of Wild Edible Plants in Southern Bénin to Identify Priority Species for Conservation1. Econ Bot [Internet]. 2011;65:381–95. Available from: https://doi.org/10.1007/s12231-011-9178-8

17. Regassa T, Kelbessa E, Asfaw Z. Ethnobotany of wild and semi-wild edible plants of Chelia District, West-Central Ethiopia. Sci Technol Arts Res J. 2015;3:122–34.

18. Schunko C, Li X, Klappoth B, Lesi F, Porcher V, Porcuna-Ferrer A, et al. Local communities' perceptions of wild edible plant and mushroom change: A systematic review. Glob Food Sec. Elsevier; 2022;32:100601.

19. Singh A, Singh RK, Kumar A, Kumar A, Kumar R, Kumar N, et al. Adaptation to social-ecological stressors: a case study with Indian jujube (Ziziphus mauritiana Lam.) growers of north-western India. Environ Dev Sustain. 2020;

20. Suwardi AB, Navia ZI, Harmawan T, Mukhar E. Ethnobotany and conservation of indigenous edible fruit plants in South Aceh, Indonesia. Biodiversitas J Biol Divers. 2020;21.

21. Tebkew M, Gebremariam Y, Mucheye T, Alemu A, Abich A, Fikir D. Uses of wild edible plants in Quara district, northwest Ethiopia: Implication for forest management. Agric Food Secur. 2018;7:1–14.

22. Tebkew M, Asfaw Z, Zewudie S. Underutilized wild edible plants in the Chilga District, northwestern Ethiopia: Focus on wild woody plants. Agric Food Secur [Internet]. 2014;3:16. Available from: http://www.agricultureandfoodsecurity.com/content/3/1/12

23. Vinceti B, Termote C, Thiombiano N, Agundez D, Lamien N. Food tree species consumed during periods of food shortage in Burkina Faso and their threats. For Syst. 2018;27:e006--e006.

24. Feyssa DH. Comparative analysis of indigenous knowledge on use and management of wild edible plants: the case of central east Shewa of Ethiopia. Ethnobot Res Appl. 2012;10:287–304.

25. Salih NKEM, Hassan Ali A. Wild food trees in Eastern Nuba mountains, Sudan: Use, diversity, and threatening factors. J Agric Rural Dev Trop Subtrop. 2014;115:1–7.
